# Postpartum Myelitis Following Dengue-Related Limbic Encephalitis

**DOI:** 10.1590/0037-8682-0472-2025

**Published:** 2026-02-06

**Authors:** Leonardo Torioni, Isidro Quispe Gonzales, Michel Elyas Jung Haziot, Arthur Moura Sarmento, Rene Leandro Magalhães Rivero, José Ernesto Vidal

**Affiliations:** 1 Instituto de Infectologia Emílio Ribas, Departamento de Neurologia, São Paulo, SP, Brasil.; 2 Instituto de Infectologia Emílio Ribas, Departamento de Infectologia, São Paulo, SP, Brasil.; 3 Instituto de Infectologia Emílio Ribas, Setor de Radiologia, Divisão de Apoio ao Diagnóstico e Terapêutica, São Paulo, SP, Brasil.; 4 Diagnósticos da América SA, Divisão de Neurorradiologia, São Paulo, SP, Brasil.; 5 Universidade de São Paulo, Instituto de Medicina Tropical, Laboratórios de Investigação Médica 49, São Paulo, SP, Brasil.; 6 Universidade de São Paulo, Faculdade de Medicina, Hospital das Clínicas, Departamento de Infectologia e Medicina Tropical, São Paulo, SP, Brasil.

A 35-week pregnant woman was diagnosed with dengue fever. Within three days, she developed confusion and seizures, requiring an emergency caesarean section and mechanical ventilation. Brain computed tomography revealed a hypodense lesion in the left temporal lobe with mass effect, compatible with limbic encephalitis(Figure 1). Cerebrospinal fluid (CSF) analysis showed 40 cells/mm³ and 80 mg/dL of protein. Empirical intravenous acyclovir (10 mg/kg every 8 h) was initiated because of the presumptive diagnosis of herpetic encephalitis. After 9 d, she was extubated but remained cognitively impaired and was discharged 11 d later with partial neurological recovery. Two days later, she was admitted to a tertiary referral hospital with fever and generalized weakness. Brain magnetic resonance imaging (MRI) demonstrated the involvement of limbic structures, hyperintensities in the cortical and subcortical brain parenchyma with subtle mass effects, cortical enhancement in these regions, and findings consistent with volume loss in the left mesial temporal lobe and insular cortex, likely resulting from previous encephalitis (Figure 2). MRI revealed demyelinating lesions in the cervical and thoracic spinal cord (Figure 3). CSF analysis showed 57 cells/mm³, more than 300 mg/dL of protein, and glucose of 42 mg/dL. Molecular tests, including those for dengue and herpesviruses, as well as metagenomic sequencing of CSF, were negative. A comprehensive panel for autoimmune encephalitis in both the blood and CSF also yielded negative results. She received pulse methylprednisolone for 5 d, followed by intravenous immunoglobulin for 5 d, with slight cognitive improvement. 

**FIGURE 1:**
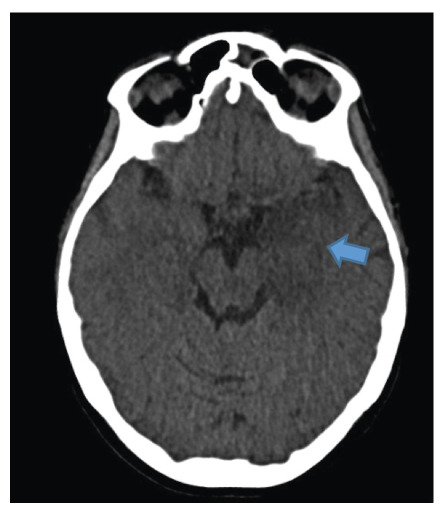
Initial CT scan of the patient. Hypodense lesion in left mesial temporal lobe with mass effect (blue arrow). Limbic encephalitis was suspected.

**FIGURE 2:**
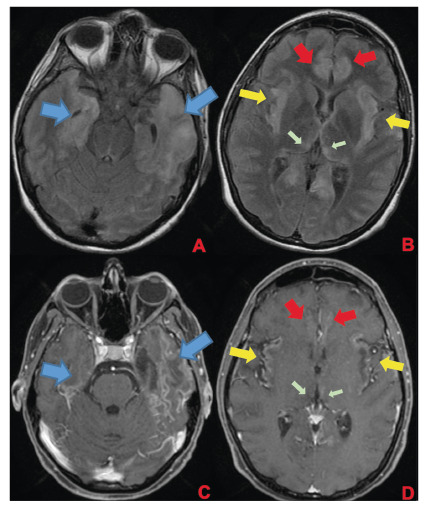
First brain MRI of the patient. In the superior row **(A and B)**, axial FLAIR images; in the inferior row **(C and D)**, axial T1 post-contrast images. Findings demonstrate encephalitis with involvement of limbic structures such as the temporal lobes (blue arrows), insula (yellow arrows), cingulate gyrus (red arrows), and medial portions of the thalami (green arrows). FLAIR images **(A and B)** show hyperintensities in cortical and subcortical brain parenchyma with a subtle mass effect, while post-gadolinium images demonstrate cortical enhancement in these regions and findings consistent with volume loss in the left mesial temporal lobe and insular cortex, likely resulting from previous encephalitis.

**FIGURE 3:**
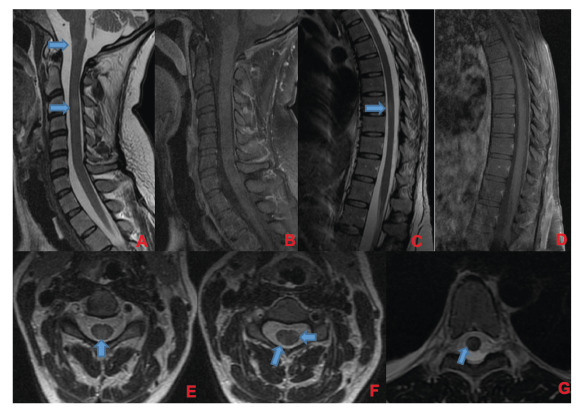
Corresponding spinal cord images of MRI shown in Figure 2. Demyelinating lesions in cervical **(A, B, E, and F)** and thoracic spinal cord **(C, D, and G)**. In A and B, sagittal plane of cervical spinal cord, showing lesions in T2-weighted image **(A)** without enhancement in T1 post-gadolinium **(B)**. There are involvement of spinal cord grey matter **(E)** and white matter **(F)** as shown in axial T2-weighted images. In **C and D**, sagittal plane of thoracic spinal cord, showing one lesion in T2-weighted image *(C)* without enhancement in T1 post-gadolinium **(D)**. This lesion was located in spinal cord white matter as shown in axial T2-weighted image in that level **(G)**. Demyelinating lesions are indicated by blue arrows.

Neurological manifestations occur in 1-5% of patients with dengue^1^. Both direct and indirect viral mechanisms contribute to dengue-associated neurological disease^2^ ^,^ ^3^. The clinical and radiological findings in our case suggest the presence of two successive neurological complications: limbic encephalitis and myelitis. The primary mechanisms include direct viral invasion and autoimmunity, respectively. This case highlights the importance of early recognition of these complications, especially during outbreaks, and the potential use of corticosteroids and immunotherapy in selected patients.

## ETHICAL STATEMENT

This study was approved by the research and ethics committees of the corresponding institutions. The study began on March/24, and the patient signed an informed consent form indicating her agreement to participate.
